# Supplementary material for: An IL-17-EGFR-TRAF4 axis contributes to the alleviation of lung inflammation in severe influenza
Source: Commun Biol. 2023 Jun 3;6:600. doi: 10.1038/s42003-023-04982-0 (PMC10239044; doi:10.1038/s42003-023-04982-0)
Supplement: Supplementary file 2 — Supplementary Information [file 42003_2023_4982_MOESM2_ESM.pdf]

Supplementary information

An IL-17-EGFR-TRAF4 axis contributes to the alleviation of lung inflammation in severe influenza

Authors: Avijit Dutta, Chen-Yiu Hung, Tse-Ching Chen, Sung-Han Hsiao, Chia-Shiang Chang, Yung-Chang Lin, Chun-Yen Lin, and Ching-Tai Huang.

Correspondence to: chingtaihuang@gmail.com

Supplementary Note 1: ***HA-specific CD4$^+$ T cell response to influenza virus infection in HA-specific CD4$^+$ TCR-transgenic 6.5 mice***

All the α/β chains of CD4$^+$ T cell receptors (TCRs) are specific for MHC Class II I-E^d restricted site of hemagglutinin (HA) from A/PR/8/34 (PR8) strain influenza virus in the 6.5 TCR-transgenic mice. Upon straight infection of 2.5×10^3 plaque-forming units (p.f.u.) of PR8 influenza virus, about 25% of the HA-specific 6.5 CD4$^+$ T cells produced archetype Th1 cytokine IFN-γ in the lungs on day 3. About 5% of the cells produced IL-17, and 1 to 2% of the cells produced both IFN-γ and IL-17 by this time. The pattern of cytokine production was altered thereafter. On day 6, less than 10% of the cells produced IFN-γ, about 25% of the cells produced IL-17, and 8 to 10% of the cells produced both IFN-γ and IL-17. The Th17 dominant response was more prominent on day 9. By this time, only 2 to 5% of the cells produced IFN-γ, more than 45% of the cells produced IL-17, and 5 to 8% of the cells produced both IFN-γ and IL-17 (Supplementary Fig. 1). This was in association with the decline of IFN-γ-producing and TNF-α-producing cells in the infected lungs during days 6 to 9 after infection (Supplementary Fig. 2).

Such a Th1 to Th17 skewed response was absent in the wild-type mice. Upon 2.5×10^3 plaque-forming units (p.f.u.) of PR8 influenza virus influenza virus infection in syngeneic wild-type mice, adoptively transferred HA-specific 6.5 CD4$^+$ T cells (2.5×10^6 naïve cells from naïve TCR-transgenic 6.5 mice) drove an HA-specific Th1 response in the lungs. On days 3, 6, and 9, there was more IFN-γ-producing than IL-17-producing HA-specific 6.5 CD4$^+$ T cells in the lungs. Moreover, the percentages of IFN-γ and IL-17 co-producing HA-specific 6.5 CD4$^+$ T cells were higher than the IL-17-producing HA-specific 6.5 CD4$^+$ T cells in the lungs on days 3, 6, and 9 after infection (Supplementary Fig. 1, 2).

Supplementary Fig. 1

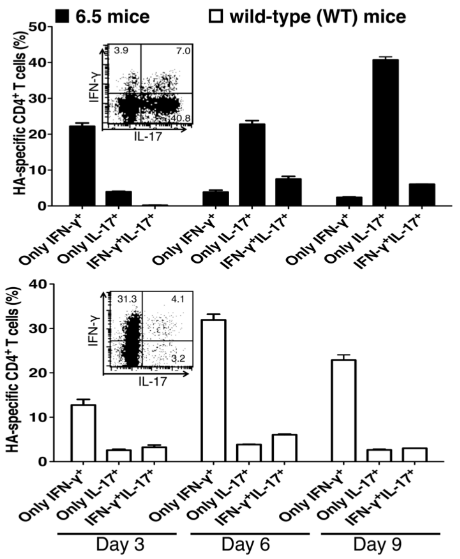

IL-17 and IFN-γ production of lung-infiltrating HA-specific CD4+ T cells in response to influenza virus infection in HA-specific TCR-transgenic 6.5 and syngeneic wild-type mice. HA-specific TCR-transgenic 6.5 and syngeneic wild-type mice were infected with 2.5×10^3 p.f.u. PR8 strain H1N1 influenza virus. Infected Thy 1.2^+ wild-type mice received adoptive transfer of 2.5×10^6 Thy 1.1^+ naïve HA-specific 6.5 $CD4^+$ T cells at the time of infection. Values are mean ± s.d. of IL-17 and IFN-γ production by the lung-infiltrating HA-specific 6.5 $CD4^+$ T cells on stated days after infection in at least 3 experiments, as detected with intracellular cytokine staining (n=6/group).

Supplementary Fig. 2

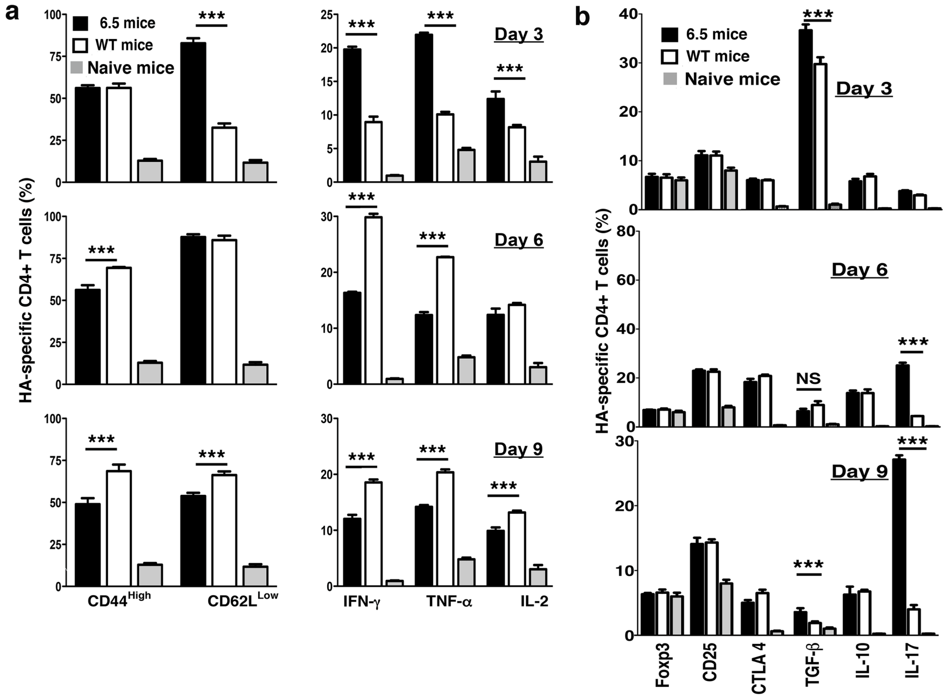

Activation profiles of lung-infiltrating HA-specific CD4$^+$ T cells in response to influenza virus infection in HA-specific TCR-transgenic 6.5 and syngeneic wild-type mice. HA-specific TCR-transgenic 6.5 and syngeneic wild-type mice were infected with 2.5×10^3 p.f.u. PR8 strain H1N1 influenza virus. Infected Thy 1.2$^+$ wild-type mice received adoptive transfer of 2.5×10^6 Thy 1.1$^+$ naïve HA-specific 6.5 CD4$^+$ T cells at the time of infection. Expression of the stated molecules of (a) activation and (b) regulatory phenotypes of lung-infiltrating HA-specific CD4$^+$ T cells were examined on stated days after infection, as detected with surface and intracellular cytokine staining. Values are means ± s.d. of at least three experiments (n=6/group; ***=p<0.0001; NS=Non-significant, p>0.05; two-tailed P values for unpaired t-test).

Supplementary Note 2: ***Decreased T-bet dominance over ROR-γt and pre-existing Th1 cell-guided Th17 response.***

With 0.5×10^6 naïve cells on day 0 and 1.5×10^6 naïve cells on day 4, Day 0 cells differentiated as Th1 cells with IFN-γ production and Day 4 cells differentiated as Th17 cells with IL-17 production on day 8 after infection. We used Thy1.1 and Thy1.2 as markers to differentiate adoptively transferred HA-specific $CD4^+$ donor cell batches from each other and from endogenous $CD4^+$ T cells in recipient mice. The T-bet was dominant over ROR-γt in the the first-batch cells, and the dominance of T-bet over ROR-γt was decreased with the excessive ROR-γt activation in the second-batch cells (Supplementary Fig. 3).

Supplementary Fig. 3

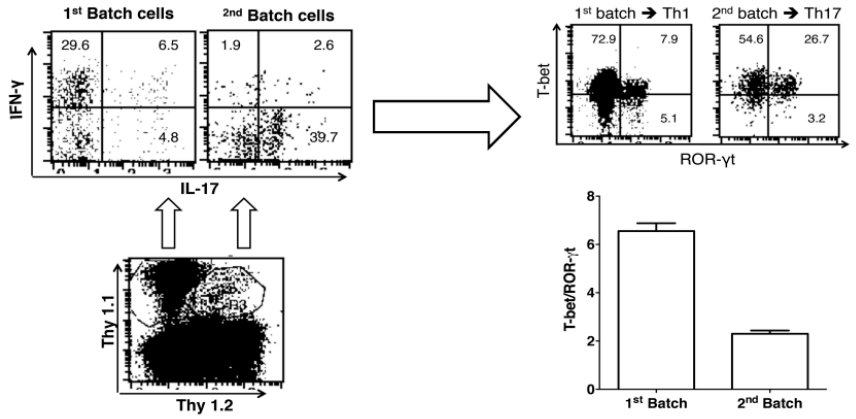

Lost T-bet dominance over ROR-γt in the Th17 response of second-batch HA-specific $CD4^+$ T cells. Wild type mice received adoptive transfer of two batches naïve HA-specific 6.5 $CD4^+$ T cells. A first batch of 0.5×10^6 Thy 1.1/Thy1.1 cells were transferred into syngeneic Thy 1.2/Thy 1.2 wild type mice at the time of 2.5×10^3 p.f.u. PR8 strain H1N1 influenza virus infection. The second batch of 1.5×10^6 Thy 1.1/Thy1.2 cells were transferred on day 4 after infection. Infected mice were killed on day 8 and lung cells were analyzed for stated parameters. Control mice

received first- or second- batch cell transfer only, with the infection. Dot-plots are representative, and other values are mean ± s.d. of at least 3 experiments (n=6/group).

Supplementary Note 3: ***IL-17 deficiency in the second-batch HA-specific CD4⁺ T cells intensifies inflammation and aggravates the disease in the two-batch adoptive transfer experiment in IL-10KO mice***

Following 2.5 × 10^3 p.f.u. PR8 strain influenza virus infection, the inflammatory response was augmented in the two-batch adoptive transfer experiment upon use of IL-17KO instead of IL-10KO second-batch cells in infected IL-10KO recipient mice. As a result of second-batch IL-17KO instead of IL-10KO cell transfer, first-batch IL-10KO donor cells produced more IFN-γ with IL-17KO than the IL-10KO second-batch cells. The IFN-γ production was also more in the IL-17KO than the IL-10 KO second-batch donor cells. With IL-17 deficiency in the second-batch HA-specific $CD4^+$ T cells, there was more virus in the lungs and more bodyweight loss in the recipient mice on Day 8 (Supplementary Fig. 4).

<u>Supplementary Fig. 4</u>

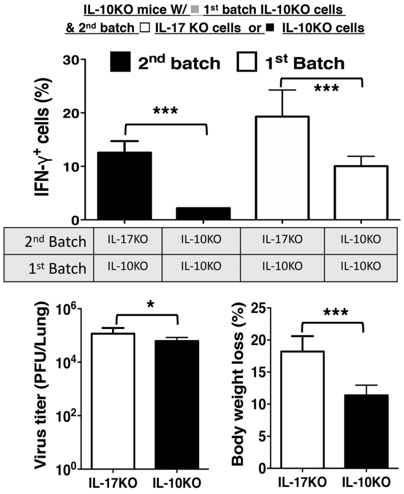

IL-17KO second-batch HA-specific $CD4^+$ T cells results in augmented inflammation an impaired virus clearance in Two-batch adoptive transfer experiment in infected IL-10KO mice. IL-10KO mice (Thy1.2/Thy1.2) received adoptive transfer of two batches naïve HA-specific 6.5 $CD4^+$ T cells with 2.5×10^3 p.f.u. PR8 strain influenza virus infection. A first batch of 0.5×10^6 IL-10KO naïve Thy 1.1/Thy1.1 HA-specific $CD4^+$ T cells were transferred at the time of infection. The second batch of 1.5×10^6 IL-10KO or IL-17KO naïve CFSE-stained Thy 1.1/Thy1.1 HA-specific $CD4^+$ T cells were transferred on day 4 after infection. Infected mice were killed on day 8. Values are mean ± s.d. of at least 3 experiments (n=6/group).

Supplementary Note 4: ***EGFR abundance than IL-17RA in the lungs of infected mice.***

We detected surface expression of EGFR and IL-17RA of lung-infiltrating $CD4^+$ T cells after infection of 2.5×10^3 p.f.u. PR8 strain influenza virus infection in TCR transgenic 6.5 mice, wild-type mice, and IL-17KO mice. We found upregulation of both the IL-17RA and EGFR on the lung-infiltrating $CD4^+$ T cell surfaces after infection. EGFR expression was highest in the 6.5 mice, and IL-17RA was higher in wild-type and IL-17KO mice (Supplementary Fig. 5).

Supplementary Fig. 5

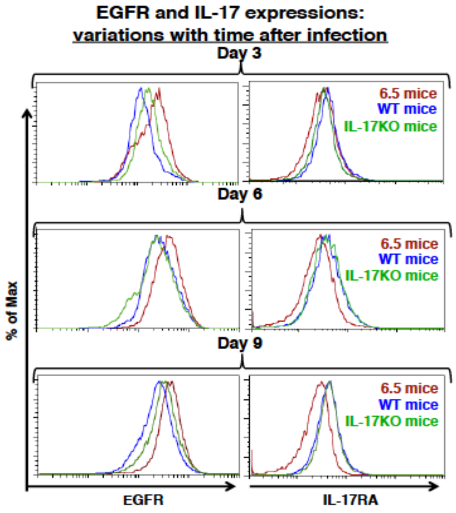

EGFR and IL-17RA expressions on the surface of lung-infiltrating $CD4^+$ T cells in infected mice. 6.5 mice, wild-type mice and IL-17KO mice were infected with 2.5×10^3 p.f.u. PR8 strain influenza virus. Non-infected healthy mice (naïve) served as controls. Histograms are representatives.
