# Supplementary material for: An IL-17-EGFR-TRAF4 axis contributes to the alleviation of lung inflammation in severe influenza
Source: Commun Biol. 2023 Jun 3;6:600. doi: 10.1038/s42003-023-04982-0 (PMC10239044; doi:10.1038/s42003-023-04982-0)
Supplement: Supplementary file 3 — Description of Additional Supplementary Files [file 42003_2023_4982_MOESM3_ESM.pdf]

Description of Additional Supplementary Files

File name: Supplemental Data 1
Description: Numerical source data for graphs and charts.
