# Supplementary material for: An IL-17-EGFR-TRAF4 axis contributes to the alleviation of lung inflammation in severe influenza
Source: Commun Biol. 2023 Jun 3;6:600. doi: 10.1038/s42003-023-04982-0 (PMC10239044; doi:10.1038/s42003-023-04982-0)
Supplement: Supplementary file 5 — Reporting Summary [file 42003_2023_4982_MOESM5_ESM.pdf]

nature portfolio

Corresponding author(s): Ching-Tai Huang

Last updated by author(s): May 15, 2023

Reporting Summary

Nature Portfolio wishes to improve the reproducibility of the work that we publish. This form provides structure for consistency and transparency in reporting. For further information on Nature Portfolio policies, see our Editorial Policies and the Editorial Policy Checklist.

Statistics

For all statistical analyses, confirm that the following items are present in the figure legend, table legend, main text, or Methods section.

n/a	Confirmed	
☐	☒	The exact sample size (*n*) for each experimental group/condition, given as a discrete number and unit of measurement
☐	☒	A statement on whether measurements were taken from distinct samples or whether the same sample was measured repeatedly
☐	☒	The statistical test(s) used AND whether they are one- or two-sided<br>*Only common tests should be described solely by name; describe more complex techniques in the Methods section.*
☒	☐	A description of all covariates tested
☒	☐	A description of any assumptions or corrections, such as tests of normality and adjustment for multiple comparisons
☐	☒	A full description of the statistical parameters including central tendency (e.g. means) or other basic estimates (e.g. regression coefficient) AND variation (e.g. standard deviation) or associated estimates of uncertainty (e.g. confidence intervals)
☐	☒	For null hypothesis testing, the test statistic (e.g. *F*, *t*, *r*) with confidence intervals, effect sizes, degrees of freedom and *P* value noted<br>*Give P values as exact values whenever suitable.*
☒	☐	For Bayesian analysis, information on the choice of priors and Markov chain Monte Carlo settings
☒	☐	For hierarchical and complex designs, identification of the appropriate level for tests and full reporting of outcomes
☒	☐	Estimates of effect sizes (e.g. Cohen's *d*, Pearson's *r*), indicating how they were calculated

Our web collection on statistics for biologists contains articles on many of the points above.

Software and code

Policy information about availability of computer code

Data collection	The majority of the data were obtained through flow cytometry. Relevant antibody names, catalog number, producer's name are included in the manuscript.
Data analysis	Flow cytometry data were Cells were acquired using FACS CALIBUR, and analysis was performed using CellQuest Pro (BD Biosciences), FACSuite (BD Biosciences), or FlowJo (Tree Star, Inc.) software. Statistical analyses were done through Graphpad Prism version 8.

For manuscripts utilizing custom algorithms or software that are central to the research but not yet described in published literature, software must be made available to editors and reviewers. We strongly encourage code deposition in a community repository (e.g. GitHub). See the Nature Portfolio guidelines for submitting code & software for further information.

Data

Policy information about availability of data

All manuscripts must include a data availability statement. This statement should provide the following information, where applicable:

- Accession codes, unique identifiers, or web links for publicly available datasets
- A description of any restrictions on data availability
- For clinical datasets or third party data, please ensure that the statement adheres to our policy

All data are presented in the manuscript. Further details can be obtained through communication to the corresponding author Prof. Ching-Tai Huang at his email: chingtaihuang@gmail.com

Human research participants

Policy information about studies involving human research participants and Sex and Gender in Research.

Reporting on sex and gender	Not applicable
Population characteristics	Not applicable
Recruitment	Not applicable
Ethics oversight	Not applicable

Note that full information on the approval of the study protocol must also be provided in the manuscript.

Field-specific reporting

Please select the one below that is the best fit for your research. If you are not sure, read the appropriate sections before making your selection.

- [x] Life sciences
- [ ] Behavioural & social sciences
- [ ] Ecological, evolutionary & environmental sciences

For a reference copy of the document with all sections, see nature.com/documents/nr-reporting-summary-flat.pdf

Life sciences study design

All studies must disclose on these points even when the disclosure is negative.

Sample size	n=6/group; each experiment was repeated for at least 3 times.
Data exclusions	No exclusion
Replication	each experiment was repeated for at least 3 times.
Randomization	For all treatment experiments, mice were first infected with influenza virus, and then randomly grouped into treated and untreated groups.
Blinding	Virus burden, lung pathology, and flow cytometry data were collected blindly, matched with mouse tag numbers to perform group-wise statistical analyses.

Reporting for specific materials, systems and methods

We require information from authors about some types of materials, experimental systems and methods used in many studies. Here, indicate whether each material, system or method listed is relevant to your study. If you are not sure if a list item applies to your research, read the appropriate section before selecting a response.

Materials & experimental systems

n/a	Involved in the study	
☐	☒	Antibodies
☐	☒	Eukaryotic cell lines
☒	☐	Palaeontology and archaeology
☐	☒	Animals and other organisms
☒	☐	Clinical data
☐	☐	Dual use research of concern

Methods

n/a	Involved in the study	
☒	☐	ChIP-seq
☐	☒	Flow cytometry
☒	☐	MRI-based neuroimaging

Antibodies

Antibodies used	Data provided in the manuscript
Validation	Data provided in the manuscript

Eukaryotic cell lines

Policy information about cell lines and Sex and Gender in Research

Cell line source(s)	Madin-Darby canine kidney (MDCK) cells were purchased from ATCC.
Authentication	We did not authenticate the cell line by ourselves
Mycoplasma contamination	Cell lines were not tested for mycoplasma contamination
Commonly misidentified lines (See ICLAC register)	Not applicable

Animals and other research organisms

Policy information about studies involving animals; ARRIVE guidelines recommended for reporting animal research, and Sex and Gender in Research

Laboratory animals	All mice were of C57BL/10.D2 genetic background, and 8-12-week-old mice of both sexes, except in experiments with aged mice, were used for experiments. The TCR-transgenic mouse line 6.5 expresses a TCR recognizing an I-Ed-restricted HA epitope (110SFERFEIFPKE120) of HA of PR8 influenza virus (generously provided by H. Von Boehmer, Harvard University, Boston, MA). The 6.5 mice were crossed with syngeneic IL17KO or IL10KO mice to generate 6.5 IL17KO TCR-transgenic, or 6.5IL10 KO TCR-transgenic mice, respectively. Syngeneic IL17KO mice were generated by breeding IL17KO mice of B6 genetic background (generously provided by Y-C. Day, Chang Gung Memorial Hospital, Taiwan) into mice with a C57BL/10.D2 genetic background for >9 generations. Syngeneic IL10 KO and 6.5 IL10 KO TCR-transgenic mice were described earlier in our previous publications. Mice were maintained under specific pathogen-free conditions.
Wild animals	Not applicable
Reporting on sex	Mice of both sexes were used in our experiments
Field-collected samples	Not applicable.
Ethics oversight	All experiments involving mice were performed in accordance with protocols approved by the “Animal Care and Use Committee” of Chang Gung Memorial Hospital (Approval number: IACUC 2016030301). The committee certified that all experiments involving mice were carried out in strict accordance with the Animal Protection Law by the Council of Agriculture, Executive Yuan, Taiwan, R.O.C., and the guidelines contained in the Guide for the Care and Use of Laboratory Animals, as promulgated by the Institute of Laboratory Animal Resources, National Research Council, U.S.A.

Note that full information on the approval of the study protocol must also be provided in the manuscript.

Dual use research of concern

Policy information about dual use research of concern

Hazards

Could the accidental, deliberate or reckless misuse of agents or technologies generated in the work, or the application of information presented in the manuscript, pose a threat to:

No	Yes	
☒	☐	Public health
☒	☐	National security
☒	☐	Crops and/or livestock
☒	☐	Ecosystems
☒	☐	Any other significant area

Experiments of concern

Does the work involve any of these experiments of concern:

No	Yes	
☒	☐	Demonstrate how to render a vaccine ineffective
☒	☐	Confer resistance to therapeutically useful antibiotics or antiviral agents
☒	☐	Enhance the virulence of a pathogen or render a nonpathogen virulent
☒	☐	Increase transmissibility of a pathogen
☒	☐	Alter the host range of a pathogen
☒	☐	Enable evasion of diagnostic/detection modalities
☒	☐	Enable the weaponization of a biological agent or toxin
☒	☐	Any other potentially harmful combination of experiments and agents

Flow Cytometry

Plots

Confirm that:

☒ The axis labels state the marker and fluorochrome used (e.g. CD4-FITC).

☒ The axis scales are clearly visible. Include numbers along axes only for bottom left plot of group (a 'group' is an analysis of identical markers).

☒ All plots are contour plots with outliers or pseudocolor plots.

☒ A numerical value for number of cells or percentage (with statistics) is provided.

Methodology

Sample preparation	Described in the manuscript
Instrument	All major instrument details are described in the manuscript
Software	Described in the manuscript
Cell population abundance	Mentioned in the manuscript
Gating strategy	Described in the manuscript

☒ Tick this box to confirm that a figure exemplifying the gating strategy is provided in the Supplementary Information.
